# GLP-1 Receptor Agonists: Beyond Diabetes—What the Neurosurgeon Needs to Know

**DOI:** 10.1227/neuprac.0000000000000098

**Published:** 2024-06-27

**Authors:** Hael Abdulrazeq, Mazen Taman, Rohaid Ali, Cody Doberstein, Patricia Sullivan, Prakash Sampath, Albert Telfeian, Ziya Gokaslan, Jared Fridley, Wael Asaad

**Affiliations:** ‡ The Warren Alpert Medical School of Brown University, Providence, Rhode Island, USA;; § Department of Neurosurgery, Rhode Island Hospital, Providence, Rhode Island, USA

**Keywords:** Glucagon-like peptide-1, Pharmacology, Perioperative care

## Abstract

**BACKGROUND AND OBJECTIVES:**

Glucagon-like peptide-1 receptor agonists (GLP-1 RAs) have gained increasing popularity since the approval of semaglutide by the United States Food and Drug Administration for chronic weight management. Significant benefits have been noted in glycemic control and cardiovascular health. However, as increasing numbers of patients are started on these medications, it is important for neurosurgeons to have knowledge of any perioperative considerations and side effects related to this class of drugs.

**METHODS:**

We performed a qualitative literature review using the PubMed and Embase databases, using the following key words: GLP-1 RAs adverse events; GLP-1 RAs and anesthesia; substance use disorders; and addiction, functional neurosurgery, nervous system rehabilitation, and spinal cord injury. Articles of relevance to perioperative management of these medications and specific benefits in the neurosurgical field were discussed.

**RESULTS:**

Recent guidance from the American Society of Anesthesiologists demonstrates the importance of tailored management of GLP-RA drugs for surgical patients. In addition, certain positive effects have been noted with relation to substance use disorders, neural protection and rehabilitation, and neurodegenerative disorders such as Alzheimer's disease.

**CONCLUSION:**

In this article, we review what the neurosurgeon needs to know about the perioperative management of GLP-1 RAs and discuss existing literature in clinical and preclinical studies for potential indications and benefits of these medications, which can influence the management of conditions treated by neurosurgeons.

**ABBREVIATIONS:**
ADE: adverse event
AUD: alcohol use disorder
CHOP: C/EBP homologous transcription factor protein
ER: endoplasmic reticulum
GI: gastrointestinal
GLP-1: glucagon-like peptide-1
GLP-1 RAs: GLP-1 receptor agonists
GRP78: glucose regulatory protein 78
HbA1c: hemoglobin A1C
PD: Parkinson disease
RIC: remote ischemic conditioning
T2DM: type 2 diabetes mellitus.

Since the approval of semaglutide by the United States Food and Drug Administration for chronic weight management, the rise in popularity of its class of diabetes medications has been fast and widespread.^1^ Patients and physicians are both learning of various benefits and off-label indications for this group of medications, referred to as glucagon-like peptide-1 receptor agonists (GLP-1 RAs). These agents have gained recent national media attention for their touted benefits of glycemic control, weight loss, and improved cardiovascular health. However, we are also beginning to learn of adverse effects, including pancreatitis, renal dysfunction, gastroparesis, and serious anesthesia complications, leading the American Society of Anesthesiologists (ASA) to release new guidance for perioperative management of these medications.^2-7^

As the number of patients on GLP-1 RAs rises, surgeons must be aware of the nuances of these drugs, and their unique perioperative risk profile. In addition, the pharmacological regulation of the GLP-1 metabolic pathway has potentially profound and widespread implications for the management of many neurosurgical conditions, potentially providing new therapeutic agents to augment our current interventions. With emerging preclinical studies showing promising results for the use of this class of drugs in both the neural protection and rehabilitation in various pathologies, including traumatic brain injury, spinal cord injury, and subarachnoid hemorrhage, the potential for clinical research and further investigation for clinical use in neurological conditions continues to expand. This literature review aims to provide neurosurgeons with current insights into the increasingly prominent GLP-1 RAs, considerations for the perioperative management and factors to consider in surgical patients, and the implications for their increasing utilization in patients seen in neurosurgical practice.

## METHODS

**FIGURE 1.**
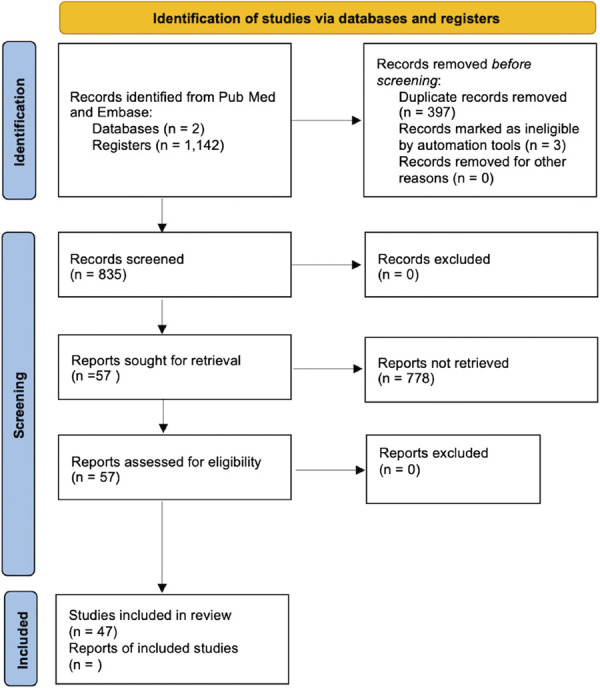
PRISMA flow diagram for new systematic reviews which included searches of databases and registers only.

We performed a literature review under the Preferred Reporting Items for Systematic reviews and Meta-Analyses (PRISMA) guidelines using the PubMed and Embase databases. The search strategy included the following keywords: "Glucagon-Like Peptide 1 Receptor Agonists"[Mesh] AND ("Neurosurgery"[Mesh] OR "Neurosurgical Procedures"[Mesh]) OR("Drug-Related Side Effects and Adverse Reactions"[Mesh]) OR (Anesthesia[Mesh]) OR ("Substance-Related Disorders"[Mesh]) OR ("Functional Neurosurgery"[Mesh]) OR ("Nervous System Rehabilitation"[Mesh]) OR ("Spinal Cord Injuries"[Mesh]) OR ("Traumatic Brain Injury"[Mesh]) OR ("Subarachnoid Hemorrhage"[Mesh]) OR ("Pituitary"[Mesh]) OR ("Surgical Procedures, Operative"[Mesh]). The search was conducted on January 5th, 2024. This review was not registered in any systematic review database. The abstracts and texts of the manuscripts from the results were reviewed. References of the articles included were also reviewed to include any studies of relevance to neurosurgeons. The inclusion criteria were studies investigating the effects and applications of GLP-1 RAs in diseases/conditions treated by neurosurgeons. Case series, retrospective studies, systematic reviews and meta-analyses, and prospective trials were included. Articles discussing surgical outcomes of patients treated for conditions other than those treated by neurosurgeons were excluded. This literature review includes studies discussing the off-label use of GLP-1 receptor agonists in neurodegenerative disorders and rehabilitation and animal studies where indicated. The results of our review were synthesized and presented in a narrative fashion in relevant sections discussing the following: GLP-1 receptor agonists: Mechanism of action, glycemic and metabolic control, cardiovascular impact, adverse events (ADEs), GLP-1 agonists and perioperative considerations, and future implications in neurosurgery: Reward pathway, neuroprotective and rehabilitative effects, and other neurosurgical considerations. Studies discussing off-label use of certain GLP-1 RAs were included in this study. Figure 1 represents a PRISMA flow chart for the screening, screening, and inclusion of studies in this review.

## RESULTS

### Mechanism of Action of GLP-1 Receptor Agonists

**FIGURE 2.**
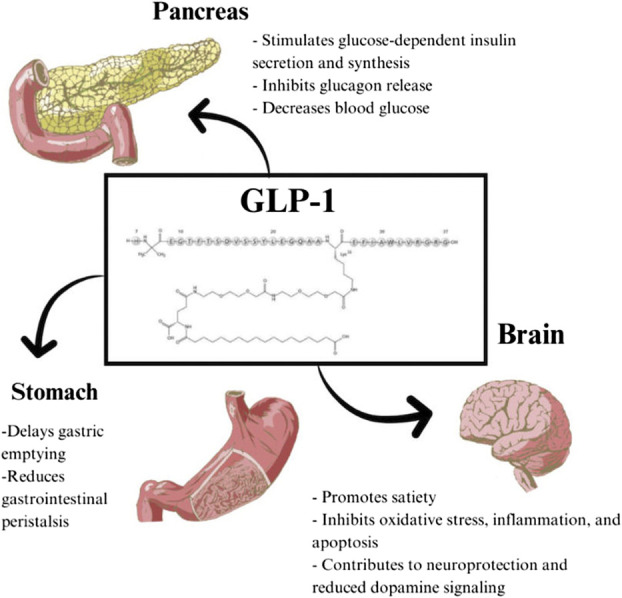
Target organ responses to GLP-1: Primary effects. GLP-1, glucagon-like peptide-1.

Glucagon-like peptide-1 (GLP-1) is an incretin hormone primarily released from neuroendocrine L cells in the ileum and colon, supplemented by a minor production from alpha cells in the pancreas and neurons in the nucleus tractus solitarius. It is released in response to the intake of food, especially carbohydrate-rich food.^8-10^ Incretins play an important role in the regulation of glucose homeostasis in response to oral glucose intake.^11^ GLP-1 is particularly important in the postprandial regulation of glycemic control by stimulating insulin release, suppressing glucagon release, delaying gastric emptying, and promoting satiety (Figure 2).^12,13^ These combined effects result in a reduction of food intake and facilitate weight loss.^13^ GLP-1 RAs work to stimulate incretin effects and have been largely used in the treatment of type 2 diabetes mellitus (T2DM) and obesity. Because of the glucose-dependent mechanism associated with activating GLP-1 receptors, the heightened insulin secretion and suppressed glucagon secretion diminish when fasting glucose levels fall below the normal range, mitigating the potential for hypoglycemia.^14^ Table offers an overview of various GLP-1 RAs, including details such as their initial dosing and pertinent considerations, including half-lives and renal adjustments.^10-21^

**TABLE.** GLP-1 RA Medication Overview

GLP-1 RA	Dosing and titration	Renal adjustments	Special considerations
Exenatide (Byetta®)^10,11^	5 mcg SQ twice daily before meals	Contraindicated in CrCl <30 mL/min	Taken 1 hour before a meal Half-life = 2.4 h
Exenatide (Bydureon)^10,12,13^	2 mg SQ once weekly	Contraindicated in CrCl <30 mL/min	Bydureon BCise formulation is a new auto-injector that does not need to be titrated or reconstituted Half-life = ∼2 wk
Liraglutide (Victoza)^10,14^ (Saxenda)^10,15^	Start with Victoza at 0.6 mg SQ daily for the first week and then escalate to 1.2 mg daily. Consider advancing to 1.8 mg daily after 4 weeks if needed Begin Saxenda at 0.6 mg SQ weekly and raise by 0.6 mg weekly until reaching a maintenance dose of 3 mg weekly	No specific adjustments, but caution in renal impairment	Liraglutide is approved for T2DM and obesity Half-life = 13 h
Dulaglutide (Trulicity)^10,16^	Initiate at 0.75-1.5 mg SQ once weekly; may increase to 3 mg once weekly after 4 weeks on the 1.5 mg dose or reach a max dose of 4.5 mg weekly after 4 weeks on the 3 mg dose	No specific adjustments, but caution in severe renal impairment	Long half-life; prefilled, single-dose pen Approved for T2DM Half-life = 5 d
Semaglutide Injectable (Ozempic)^10,17^ (Wegovy)^10,18^	Initiate Ozempic at 0.25 mg SQ once weekly. After 4 weeks, escalate to 0.5 mg once weekly. If additional glycemic control is required after at least 4 weeks, increase to 1 mg once weekly Begin Wegovy at 0.25 mg SQ once weekly. Adjust the dose every 4 weeks until reaching a maintenance dose of 1.7 mg or 2.4 mg once weekly	No specific adjustments, but caution in severe renal impairment	Semaglutide is approved for T2DM and obesity Demonstrated efficacy in reducing cardiovascular risk Half-life = ∼1 wk
Semaglutide (Rybelsus)^10,19^	Start with 3 mg PO daily for 30 days and then escalate to 7 mg daily. If necessary, titrate up to 14 mg daily	No specific adjustments, but caution in severe renal impairment	First oral GLP-1 RA Taken with or without food Half-life = ∼1 wk
Lixisenatide (Adlyxin)^10,20^	Initiate with 10 mcg SQ once daily for 14 days and then escalate to 20 mcg once daily	No specific adjustments, but caution in severe renal impairment	In combination with Lantus (Soliqua) given once daily SQ Half-life = 3 h
Tirzepatide (Mounjaro)^10,21^	Begin with 2.5 mg SQ once weekly and then elevate to 5 mg once weekly after 4 weeks. Increment by 2.5 mg every 4 weeks, up to a maximum dose of 15 mg weekly	No specific adjustments, but caution in renal impairment	Dual receptor agonist for glucose-dependent insulinotropic polypeptide (GIP) and GLP-1 Superior efficacy in T2DM and obesity when compared with semaglutide^22^ Half-life = ∼5 d

GLP-1, glucagon-like peptide-1; GLP-1 RA, GLP-1 receptor agonist; SQ, subcutaneously.

### Glycemic and Metabolic Control

The efficacy of GLP-1 RAs in glycemic control highlights their primary role in diabetes management.^15-22^ GLP-1 RAs reduce glycated hemoglobin (HbA1c: Hemoglobin A1C) with low hypoglycemia risk and lead to clinically relevant reductions in weight, body mass index, and waist circumference in overweight or obese individuals, with or without diabetes.^16,17^ These agonists, alongside sodium-glucose cotransporter-2 inhibitors, reduce glycemic variability significantly and show promising reductions in diabetes-related microvascular complications, especially nephropathy.^18,19^ Given the association between elevated HbA1c levels and increased risks of surgical wound infections and postoperative glycemic variability, preoperative administration of GLP-1 agonists could improve glycemic control and surgical outcomes.^20-22^ Furthermore, GLP-1 RAs may have effects on levels of different hormones, and its effects on the endocrine system can have implications for patients with pituitary gland lesions that neurosurgeons need to be aware of. For example, GLP-1 RAs have been shown to be associated with an increase in growth hormone release in healthy patients.^23^ Broadly, GLP-1 RAs activate neural signaling from the hypothalamus, resulting in increased secretion of neuropeptides, such as the gonadotropin releasing hormone, and various downstream effects on the hypothalamic-pituitary axis.^24^ These complex interactions may be necessary to understand in patients on GLP-1 RAs with dysfunction of the hypothalamic-pituitary axis because of other conditions such as pituitary adenomas.

### Cardiovascular Impact

Beyond glycemic control, GLP-1 RAs exhibit a significant impact on cardiovascular health. Trials have demonstrated significant reductions in major adverse cardiovascular events,^15,25-30^ systolic blood pressure, and triglyceride levels.^15,31^ Experimental studies further support their ability to mitigate oxidative stress, endothelial dysfunction, and cardiac hypertrophy.^32^ While metformin and lifestyle modification are the preferred first-line treatments in patients with T2DM and an elevated HbA1c, GLP-1 RAs are now a preferred second-line therapy and are even recommended first-line agents in patients with known atherosclerotic cardiovascular disease, high cardiovascular risk, or chronic kidney disease.^33,34^

### Adverse Events (ADEs)

Pancreatitis is the most feared ADE associated with the use of GLP-1 RAs, with increased odds of pancreatitis for GLP-1 RA users compared with other classes of glucose-lowering agents.^3,4^ While mild gastrointestinal (GI) side effects like nausea, vomiting, diarrhea, and abdominal discomfort are reported and tend to improve over time,^5^ there is also potential for other severe ADEs, such as gastroparesis and GI obstruction, which often require hospitalization, particularly in the context of surgical interventions.^6^

GLP-1 RA preclinical rodent studies demonstrate an increase in thyroid C-cell neoplasm occurrence which has led to a Food and Drug Administration black box warning.^35^ Consequently, these agents are contraindicated in patients with a personal or family history of medullary thyroid cancer or those diagnosed with multiple endocrine neoplasia syndrome type 2. Another noteworthy side effect is renal dysfunction, particularly with liraglutide and exenatide. These agents are associated with instances of acute renal failure and exacerbation of chronic renal failure.^7^

### GLP-1 Agonists and Perioperative Considerations

The ASA recently issued recommendations for the preoperative management of GLP-1 RAs for patients undergoing nonemergent surgical procedures.^2^ Specifically, for patients on a daily dosing regimen, the ASA recommends withholding a dose on the day before the procedure.^2,36^ For those following a weekly dosing schedule, it is recommended to hold the medication for a week before the scheduled surgery.^2,36^ Some physicians have taken a cautious approach by advising patients to discontinue GLP-1 RAs a week before surgery because of concern for gastroparesis, which may increase the risk of aspiration during the procedure.

On the day of the procedure, if patients experience GLP-1 RA–associated symptoms such as severe nausea, vomiting, retching, abdominal pain, or bloating, it is recommended to consider delaying the elective procedure because of the risk of aspiration.^2,36^ In situations where patients have not experienced GI symptoms but have not adhered to the recommended medication hold, the ASA suggests proceeding with “full stomach” precautions, treating the patient as if they have a high risk for aspiration. This precautionary approach may involve the assessment of gastric volume using ultrasound. If the ultrasound indicates a full stomach or produces inconclusive results, surgeons should consider delaying the procedure or managing the patient as a “full stomach.”^2,36^

Although specific guidance on the precise timing of reinitiating GLP-1 RAs postoperatively is not explicitly outlined, it may be reasonable to hold these medications in the immediate postoperative period and resume with a similar approach as other diabetes medications that pose a low risk of hypoglycemia.^37^ This approach involves monitoring changes in renal function and holding GLP-1 RAs that are typically taken with food until the patient can tolerate an oral diet.^37^ Moreover, in alignment with clinical recommendations, initiating sliding scale insulin on the day of surgery and targeting an in-hospital blood glucose level of <180 mg/dL may be considered, with potential inclusion of medications with a low risk of hypoglycemia on postoperative day 3.^37^

### Reward Pathway

While the primary application of GLP-1 agonists has been in management of diabetes and obesity, numerous investigations have begun to explore potential implications for addiction and psychiatric disorders.^38-40^ GLP-1 produced in the nucleus tractus solitarius with target receptors in neurons, astrocytes, and microglia plays a pivotal role in modulating responses to alcohol.^41^ Preclinical research, particularly in rodents and nonhuman primates, demonstrates a significant reduction in the intake of alcohol and drugs of abuse following administration of GLP-1 RAs.^40,42-44^ Rodent studies indicate that GLP-1 RAs suppress various alcohol-related effects, including hyperlocomotion, dopamine release, alcohol-seeking behaviors, and relapse drinking in male rodents.^38,45^ Clinical studies suggest that GLP-1 RAs may have a role in reducing alcohol consumption, especially in obese individuals with alcohol use disorder.^38,42^ Furthermore, observational studies indicate a decrease in alcohol-related events, defined as hospitalizations with a primary diagnosis of alcohol use disorder, registered treatments for alcoholism, or the acquisition of specific medications linked to alcohol withdrawal or dependence, when comparing GLP-1 receptor agonists with dipeptidyl peptidase 4 inhibitors.^46^ In individuals addicted to cocaine, reduced GLP-1 levels following cocaine injection are associated with increased cocaine intake and anxiety, while exenatide-4 supplementation decreases cocaine-induced preference and dopamine release.^47,48^ Moreover, exenatide-4 exhibits a mitigating effect on abnormal mobility and dopamine release, as well as locomotor behavior associated with amphetamine use.^48^ The impact shown by this class of drugs on various substance use disorders suggests a potential role in reducing postoperative opioid use in neurosurgery patients with chronic pain undergoing surgery. Further studies comparing narcotic use in neurosurgical patients on GLP-1 RAs vs those who are not will be necessary to quantify this effect.

The potential relevance of GLP-1 RAs to functional neurosurgery will require a better understanding of their clinical effects and mechanisms of action, particularly in the management of substance use disorders. As the role of deep brain stimulation in treating these disorders advances, attention should be paid to the role that GLP-1 RAs might offer as an adjunctive or alternative treatment, potentially mitigating the need for neurosurgical intervention.^49-52^ While evidence suggests central mediation, possibly through dopamine signaling, the precise neurocircuit-level mechanisms are yet to be fully elucidated.^38,42^

### Neuroprotective Effects

Early preclinical work suggests several potential beneficial effects of targeting the GLP-1 metabolic pathway. For example, animal studies on GLP-1 RAs have shown in animal studies to have potential neuroprotection within the central and peripheral nervous systems. Specifically, stimulation of GLP-1 receptors is associated with increased proliferation of progenitor cells in the dentate gyrus, a region of the brain associated with learning and memory, and a positive impact on synaptic plasticity.^53,54^ GLP-1 RAs may also offer neuroprotection by preventing apoptosis induced by hypoxia or chronic hyperglycemia products, suggesting a role in regulating brain glucose levels.^55-57^ These findings potentially have significant research and clinical applications in patients with various types of brain injury, including traumatic brain injury, ischemia, and hemorrhagic stroke. Specifically, animal models of traumatic brain injuries have demonstrated positive effects in reducing the inflammatory response in this type of injury, with potential positive effects on recovery.^58-61^ The preclinical studies presented herewith may represent early steps for studying the neuroprotective effects of GLP-1 RAs in neurosurgical patients.

In addition, GLP-1 RAs may reduce accumulation of amyloid precursor protein, improve cognitive function, increase proliferation of neuronal progenitor cells, and attenuate neuronal damage postischemia, in Alzheimer disease and Parkinson disease (PD).^62-66^ Liraglutide, for example, was observed to reduce amyloid precursor protein accumulation and neuroinflammation, leading to improved cognitive outcomes.^67-69^ In patients with PD, exenatide has shown promise in mitigating cognitive decline.^70^ A meta-analysis reported that exenatide usage extended lifespan, prevented cognitive impairment, and improved quality of life in patients with PD, as indicated by increased Unified Parkinson's Disease Rating Scale scores and cognitive test scores.^71-73^ Studies with exenatide in PD patients support its potential for treating motor dysfunction, leading to decreases in nonmotor symptoms as well as motor complications.^71^ These recent trials have shown some promise for the use of GLP-1 RA in patients with neurodegenerative conditions and movement disorders, which may have implications for their use as augmentative therapies along with neurosurgical treatments such as deep brain stimulation, as well as high intensity focus ultrasound. Not only can they have effects on the efficacy of these neurosurgical interventions but may also influence the eligibility of patients with cognitive impairment for these procedures, though future research is needed to study these effects in the context of neurosurgery.

### Rehabilitative Effects

GLP-1 RAs, such as exenatide, may even contribute to the rehabilitation and functional improvement of spinal cord injuries and nerve degeneration. In a rat model of SCI, subcutaneous administration of exenatide immediately after injury and again 7 days later resulted in a significant improvement in hindlimb function without inducing hypoglycemia.^74^ This improvement was associated with a modulation of the endoplasmic reticulum (ER) stress response, reflected in suppressed levels of C/EBP homologous transcription factor protein (CHOP), a proapoptotic factor, and increased expression of glucose regulatory protein 78, a chaperone protecting against ER stress.^74^ The subsequent reduction in ER stress led to a substantial decrease in tissue damage and increased survival of oligodendrocyte progenitor cells.^74^ In a separate study involving peripheral nerve injury, repeated intraperitoneal injections of exendin-4, a long-acting analog of GLP-1, promoted recovery after crush nerve injury, improving functional, electrophysiological, and morphological parameters associated with nerve regeneration.^75^ These findings highlight the potential of GLP-1 receptor agonists in mitigating secondary injury processes, enhancing neural regeneration, and improving functional outcomes in both spinal cord and peripheral nerve injuries. As such, these neuroprotective and rehabilitative effects may have significant implications for the field of neurosurgery, potentially influencing treatment approaches and strategies for patients with such injuries.

These studies illustrate the evolving landscape of GLP-1 RAs and their clinical applications in neurodegenerative conditions and spinal cord and peripheral nerve injury, opening avenues for further research and clinical applications.

### Impact on Obesity and Associated Surgical Complications

Obesity is associated with a heightened complication profile and increased odds of unfavorable outcomes in various neurosurgical procedures.^76^ In patients undergoing cranial or spinal procedures, an elevated body mass index emerges as a substantial risk factor for surgical site infection, venous thromboembolism, major medical complications, prolonged surgical duration, and heightened financial costs.^76-80^ Considering the link between obesity and spinal diseases, studies have demonstrated that overweight and obese individuals are more likely to develop lower back problems and degenerative disk disease.^76,81^ This association further underscores the significant impact of obesity on patient outcomes in neurosurgical practice and emphasizes the need for careful consideration of surgical approaches in this patient population. Recognition of the potential of GLP-1 RAs to address obesity-related concerns, along with their potential for enhanced anti-inflammatory and neuroprotective effects, again indicates the importance of additional research on integration of this drug class into the perioperative care of neurosurgical patients.

### Vascular Disease

GLP-1 RAs have shown potential use in the primary prevention of stroke. A pooled analysis of 5 randomized placebo-controlled trials demonstrated a significant 13% reduction in the risk of total stroke with GLP-1 RAs compared with placebo, without significant heterogeneity between trials.^25-27,82-84^ Notably, weekly subcutaneous semaglutide at 2.4 mg demonstrated superiority over placebo in reducing the incidence of cardiovascular death, nonfatal myocardial infarction, and nonfatal stroke in individuals with preexisting cardiovascular disease, obesity, and without diabetes.^30^ Moreover, GLP-1 RAs exhibit protective effects on pancreatic β-cells against glucose toxicity and show promise for arteriosclerosis prevention, particularly in the early stages of T2DM.^85^ One study suggests a potential interconnection between remote ischemic conditioning (RIC) and GLP-1 RAs in neuroprotection against ischemic stroke.^86^ RIC involves inducing brief, controlled episodes of ischemia and reperfusion to induce protective mechanisms which create tolerance to subsequent ischemic events in the target organ.^86^ RIC and systemic GLP-1R agonist administration led to an 80% reduction in cerebral infarct size and improved neurological scores in a rat model.^86^ Analysis of GLP-1 receptor expression in the cerebral cortex and ex vivo experiments demonstrated a potent dilatory effect on cortical arterioles, suggesting the potential for improved brain perfusion with the administration of GLP-1 RAs.^86^ Furthermore, GLP-1 RAs demonstrate protective effects in stroke models, as indicated by a reduction in infarct volume and improved neurological symptoms and prognosis.^87,88^ Of note, preclinical models of subarachnoid hemorrhage in rats have shown promising results in preserving the blood brain barrier and reducing rates of neuronal apoptosis, thereby working to decrease early brain injury.^89-91^

**FIGURE 3.**
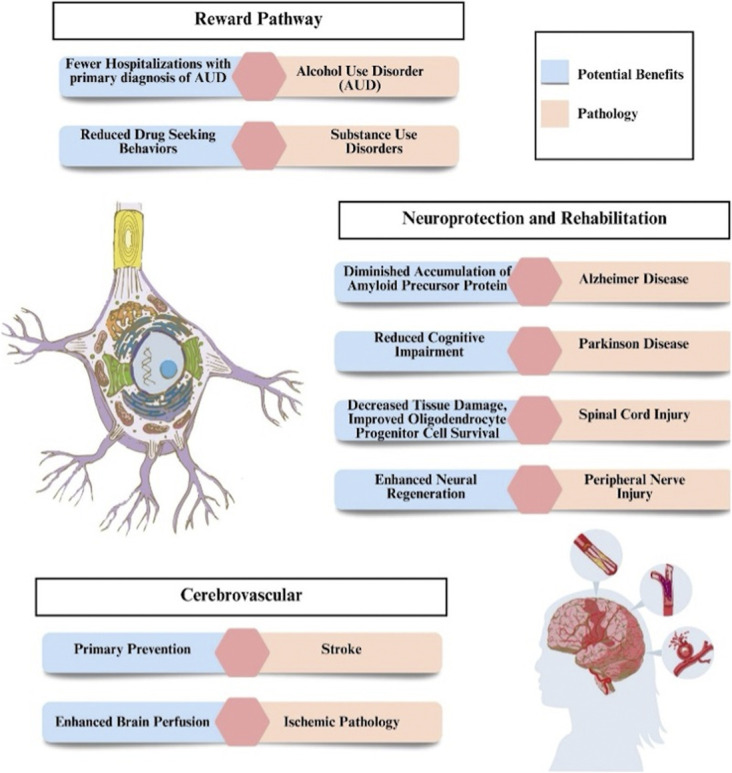
Potential implications of GLP-1 receptor agonists on neurosurgery based on clinical and preclinical studies.

Using preclinical models, Figure 3 outlines the prospective influence of GLP-1 RAs on the future landscape of neurosurgery and underscores their potential implications. While experimental evidence supports the neuroprotective potential of GLP-1 activation, further research is necessary to expand this work into the clinical setting and uncover the full therapeutic potential of GLP-1 RAs in stroke management and prevention.

### Limitations

Current evidence on the use of GLP-1 RAs for the indications discussed in this review remains in its early stages, and represents predominantly preclinical studies in the fields of neural rehabilitation and recovery. Further prospective clinical trials are needed to better understand the benefits and risks of using this class of drugs in various disorders affecting neurosurgical patients.

## CONCLUSION

GLP-1 RAs play a pivotal role in various medical domains, ranging from glycemic control to cardiovascular health, neuroprotection, and potential applications in neurosurgery. However, caution is necessary because of the risk of ADEs, such as pancreatitis and renal dysfunction, and their potential implications in the perioperative setting, where careful management is required.

GLP-1 RAs may have intriguing future implications for neurosurgery. The exploration of GLP-1 RAs in addiction and psychiatric disorders, particularly their potential in reducing alcohol and substance abuse, opens new avenues for research and intervention in the domain of functional neurosurgery. The neuroprotective effects observed in neurodegenerative conditions such as Alzheimer disease, PD, and stroke suggest potentially enhanced recovery and better outcomes for patients with devastating neurological diseases. Likewise, the neuroprotective and rehabilitative effects observed in spinal cord injury and nerve degeneration potentially offer new treatment strategies. In addition, GLP-1 RAs may offer a potent tool in managing the numerous increased risks of spine surgery in obese patients. As GLP-1 RAs gain recognition for their potential benefits beyond glycemic control and metabolic regulation, their broader applications warrant a more comprehensive understanding to optimize patient safety and outcomes in the dynamic field of neurosurgery.
